# Identifying high school risk factors that forecast heavy drinking onset in understudied young adults

**DOI:** 10.1016/j.dcn.2024.101413

**Published:** 2024-06-26

**Authors:** Qingyu Zhao, Magdalini Paschali, Joseph Dehoney, Fiona C. Baker, Massimiliano de Zambotti, Michael D. De Bellis, David B. Goldston, Kate B. Nooner, Duncan B. Clark, Beatriz Luna, Bonnie J. Nagel, Sandra A. Brown, Susan F. Tapert, Sonja Eberson, Wesley K. Thompson, Adolf Pfefferbaum, Edith V. Sullivan, Kilian M. Pohl

**Affiliations:** a Department of Radiology, Weill Cornell Medicine, New York, NY, USA; b Department of Radiology, Stanford University, Stanford, CA, USA; c Center for Health Sciences, SRI International, Menlo Park, CA, USA; d Department of Psychiatry and Behavioral Sciences, Duke University School of Medicine, Durham, NC, USA; e Department of Psychology, University of North Carolina Wilmington, Wilmington, NC, USA; f Department of Psychiatry, University of Pittsburgh, Pittsburgh, PA, USA; g Departments of Psychiatry and Behavioral Neuroscience, Oregon Health & Science University, Portland, OR, USA; h Department of Psychology, University of California San Diego, La Jolla, CA, USA; i Department of Psychiatry, University of California San Diego, La Jolla, CA, USA; j Laureate Institute for Brain Research, Tulsa, OK, USA; k Department of Psychiatry and Behavioral Sciences, Stanford University, Stanford, CA, USA

**Keywords:** Alcohol, Forecasting, Young adult, Adolescence, College

## Abstract

Heavy alcohol drinking is a major, preventable problem that adversely impacts the physical and mental health of US young adults. Studies seeking drinking risk factors typically focus on young adults who enrolled in 4-year residential college programs (4YCP) even though most high school graduates join the workforce, military, or community colleges. We examined 106 of these understudied young adults (USYA) and 453 4YCPs from the National Consortium on Alcohol and NeuroDevelopment in Adolescence (NCANDA) by longitudinally following their drinking patterns for 8 years from adolescence to young adulthood. All participants were no-to-low drinkers during high school. Whereas 4YCP individuals were more likely to initiate heavy drinking during college years, USYA participants did so later. Using mental health metrics recorded during high school, machine learning forecasted individual-level risk for initiating heavy drinking after leaving high school. The risk factors differed between demographically matched USYA and 4YCP individuals and between sexes. Predictors for USYA drinkers were sexual abuse, physical abuse for girls, and extraversion for boys, whereas 4YCP drinkers were predicted by the ability to recognize facial emotion and, for boys, greater openness. Thus, alcohol prevention programs need to give special consideration to those joining the workforce, military, or community colleges, who make up the majority of this age group.

## Highlights

• Young adults who joined 4-year residential colleges were likely to initiate heavy drinking during college years.
• Young adults who joined the workforce, military, or community colleges increased drinking after college age.
• Risk factors during high school that forecast heavy drinking onset in young adulthood differ between the two career paths.

## 1 Introduction

The cost of heavy alcohol use is extensive, with respect to life achievement, family interaction, and physical and mental health (White and Hingson, 2013). Epidemiological studies have consistently found heavy alcohol drinking to be most prevalent among young adults (Hasin et al., 2007), with 29.2 % of those ages 18–25 years reporting past-month binge drinking and 14.9 % meeting criteria for past-year alcohol use disorder (AUD) (NIAAA, 2023, SAMHSA, 2023). While substantial research has been dedicated to young adults in 4-year residential college programs (4YCP) (NIAAA, 2022), the National Institute on Alcohol Abuse and Alcoholism recently highlighted the need for prevention and screening research on “understudied” young adults (USYA) ages 18–29 years who are not enrolled in 4-year colleges or universities (NIAAA, 2022). These individuals account for the majority of the young adult population (NCES, 2022), commonly serving in the military, joining the workforce, or enrolling in local community colleges after high school.

The lack of attention paid to these understudied young adults has likely resulted from challenges associated with tracking the more diverse working and living situations of the USYA cohort relative to the 4YCP young adults compared with greater accessibility to youth residing on university campuses and surrounds (Carter et al., 2010). In fact, differences in drinking behaviors between the two cohorts may already be apparent before college (Johnston et al., 2023), as USYA individuals reported greater quantities of alcohol consumption during high school from those who subsequently attended college (Timberlake et al., 2007). Regarding psychological experiences and constructs, USYA generally reported a higher number of childhood traumatic events, greater family drinking history, and higher depressive symptoms during adolescence than 4YCP (Blanco et al., 2008, Dennis, 2021). Still unknown, however, is whether the risk factors among no-to-low drinking high school students for predicting drinking onset in early adulthood are different between USYA and 4YCP.

Sex is another likely factor distinguishing the pathway from alcohol-naïve high school students to heavy drinking young adults (Patrick et al., 2023). Although rates of heavy alcohol consumption are now similar for male and female youth (White, 2020), sex-specific developmental factors underlie sex-specific risk factors for drinking onset. For example, more male than female youth evidence emergent externalizing behaviors or traits, such as impulsivity and sensation seeking (Dir et al., 2017), whereas more female than male youth express internalizing symptoms, such as anxiety and depression (Dir et al., 2017). These sex-related phenotypes emerging during adolescence have the potential to moderate the risk level for initiating heavy drinking in young adulthood but may be expressed differentially with respect to timing, living, and working environment. Consequently, sex-specific risk factors forecasting drinking onset in USYA may differ from those observed in the 4YCP cohort.

**Fig. 1:**
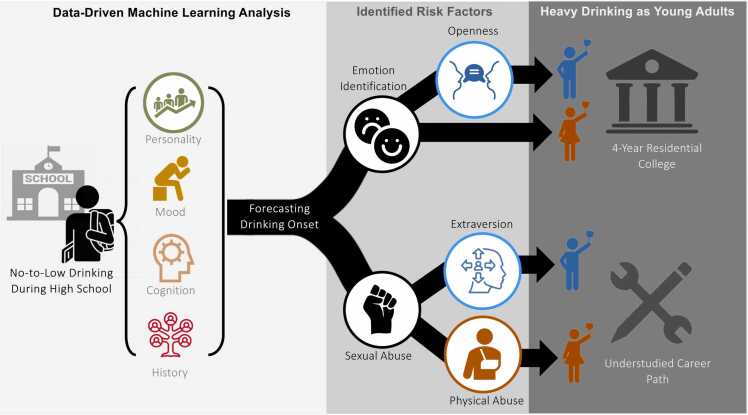
Based on mental health measurements recorded during high school, machine learning analysis identified significant risk factors for forecasting drinking onset post high school that differed by sex and early career choice.

Herein, we tracked the drinking trajectories of 106 USYA and 453 4YCP participants in the National Consortium on Alcohol and NeuroDevelopment in Adolescence (NCANDA) study (Brown et al., 2015). These participants had a no-to-low drinking history before age 18 years and participated in 1–8 annual follow-up assessments through young adulthood. We then used machine learning to forecast which adolescents would initiate heavy drinking in young adulthood based on data recorded during high school (Fig. 1). As portrayed in Fig. 1, our data-driven analysis identified adolescent risk factors from an array of measurements (capturing mood, personality, history, and cognition) that differentially forecast drinking onset in each sex and post-high-school career choice.

## 2 Material and methods

### 2.1 Participants

The NCANDA cohort (Brown et al., 2015) comprises 831 participants, ages 12–21 years at baseline who were recruited across five collection sites in the US and assessed yearly on psychobiological measures. Based on self-reported alcohol use history collected throughout the first 8 years of the study (Data Releases NCANDA_NIAAADA_BASE_V01, NCANDA_NIAAADA_01Y_V01 to NCANDA_NIAAADA_08Y_V01 available via https://nda.nih.gov/edit_collection.html?id=4513), drinking levels of participants were defined based on the youth-adjusted Cahalan score on a scale of 0–3 (Cahalan et al., 1969, Pfefferbaum et al., 2018). College status was defined by the combination of three variables in the youth report: “What is the highest grade (or year) of regular school that you have completed?”, “What is the highest degree you have earned?”, and “What is your current education involvement?”. Those who indicated participating 4-year colleges or earned Bachelor’s or higher degrees were categorized as 4YCP. The remaining participants who reported participating community colleges or reported “done with school” with highest grade no greater than 12th grade were categorized as USYA.

To identify risk factors for predicting drinking onset at or after age of 18, our study focused on 559 participants (Table 1, Table S3) who remained no-to-low drinkers before age 18 years (youth-adjusted Cahalan score = 0, minimum age 12 years) and had known college and drinking status in young adulthood (maximum age 26 years, number of visits per subject = 7.2±1.8). Based on the combination of the highest grade, highest earned degree, and current education involvement recorded in youth report, 453 participants went to 4-year residential college programs (4YCP, leading to Bachelor’s degrees) after age 18 years, and 272 of those participants had graduated from college. Among the remaining 106 USYA participants who did not go to 4YCP, 67 attended community colleges with 38 of them having a full-time job after age 18 years. The remaining 39 USYA participants did not attend any type of college with 18 of them having a full-time job and 4 joining the military during that age range. Of these 106 USYA participants, 30 drank heavily during young adulthood (i.e., youth-adjusted Cahalan score > 1 in at least one visit at age 18–26 years), while 258 of the 453 4YCP participants heavily drank. There was no significant difference in the number of visits between non-heavy (youth-adjusted Cahalan score ≤ 1) and heavy drinkers in USYA or 4YCP. Supplement Methods further describe the participants and drinking criteria.

**Table 1:** Demographic information of the understudied young adults (USYA) in the NCANDA study, who were no-to-low drinkers before age 18 years and attended community college or did not go to college after high school. Their peers who enrolled in 4-Year residential College Programs (4YCP) were matched with respect to sample size, socioeconomic status, race, and site information to the USYA cohort.

	USYA	4YCP (Matched)	4YCP (All)
N (male/female)	106 (49/57)	106 (38/68)	453 (209/244)
Age (in years) at baseline	15.7±2.5*	16.5±2.6*	16.9±2.6*
Number of visits	7.1±1.7	7.5±1.6	7.2±1.8
Heavy drinkers past 18 years (%)	30 (28 %)*	62 (58 %)*	258 (57 %)*
Race (%)			
Caucasian	61 (58 %)	70 (66 %)	349 (77 %)*
African-American	34 (32 %)	20 (19 %)	42 (9 %)*
Asian	5 (5 %)	8 (7.5 %)	44 (10 %)*
Others	6 (6 %)	8 (7.5 %)	18 (4 %)*
Site (%)			
University of Pittsburgh	18 (17 %)	15 (14 %)	74 (16 %)*
SRI International	8 (8 %)	12 (11 %)	70 (15 %)*
Duke	21 (20 %)	16 (15 %)	112 (25 %)*
Oregon Health & Science University	22 (21 %)	29 (28 %)	88 (19 %)*
University of California San Diego	37 (35 %)	34 (32 %)	109 (24 %)*
Socioeconomic status	14.7±2.2	15.0±2.4	17.2±2.2*

* p<0.05 difference between USYA and 4YCP

### 2.2 Alcohol drinking trajectory of 559 young adults at or after age 18 years

Alcohol use of a participant was quantified by the log of the number of drinking days in the past year measured at each visit (Zhao et al., 2021). A quadratic mixed effects model with random intercepts (Laird and Ware, 1982) was used to model the group-level drinking trajectory for the 453 4YCP and 106 USYA participants at age 18 years or older (total number of visits = 2623). Other fixed-effect covariates included sex, race/ethnicity, data collection site, socioeconomic status, college group (4YCP or USYA), and the interaction between age and college group. An interaction with p<0.05 would suggest that difference in drinking between 4YCP and USYA significantly varies with age.

### 2.3 Mental health measurements

Prediction of the onset of heavy alcohol drinking after age 18 years was based on selected measurements capturing history, personality, mood, and cognition averaged across all visits before age 18 years. Specifically, historical factors included family drinking history and self-reported traumatic experiences captured by the five subscales of the Childhood Trauma Questionnaire (CTQ, (Bernstein et al., 1994)). Depressive symptoms were measured by the Center for Epidemiologic Studies Depression Scale (CES-D-10, (Radloff, 1977)) score. Personality traits were captured by the five subscales of UPPS-P Impulsive Behavior Scale (Cyders et al., 2014) and five subscales of Ten-Item Personality Inventory (TIPI) (Nunes et al., 2018). Neuropsychological scores included the overall mean and standard deviation of the response time in the Stroop task (Schulte et al., 2020), the median response time for the correct identifications of 5 emotions in the Penn Emotion Recognition Test (Gur et al., 2002), and the true positive rate and Median Response Time for All Correct Responses in the Short Fractal N-back Test. For each of the above 27 factors, the values were averaged across visits before age 18 years, so that each participant was described by a 27-dimensional feature vector. The proportion of missing values ranged from 0 % (family history) to 33 % (Stroop test scores) for the 27 factors. The imputation strategy and procedures for removing effects from confounding variables in Table 1 are described in Supplement Methods.

### 2.4 Forecasting heavy drinking in 212 young adults

Confined to the 106 USYA participants who were no-to-low drinkers before age 18 years, a machine learning classifier predicted whether participants initiated heavy drinking or not at age 18 years or older from the 27 mental health factors. To accommodate the relatively small sample size, we chose our classifier to be a linear Support Vector Machine (SVM, as implemented in *fitcsvm* of Matlab 2020b with the default parameter setting), which has been shown to be more suitable for small dataset analysis than non-linear models (Hsu and Lin, 2002) and produced more accurate predictions than the logistic classifier LASSO (Friedman et al., 2010) (see Supplement Method). The SVM was evaluated by a Leave-One-Out cross-validation to maximize the training sample size (Kohavi, 1995). As such, the classifier produced a continuous prediction score for each USYA participant. A positive score indicated that the participant was classified as initiating heavy drinking at or after age 18 years and a negative score indicated no heavy drinking (see Supplement Methods for detailed interpretation).

Accuracy of the binary classification outcome was quantified by Balanced Accuracy (BAcc) (Park et al., 2018), area under the ROC curve (AUC), and the F1-score (Supplement Methods). Sex differences in model prediction were tested by two approaches. The first approach measured the BAcc separately in male and female participants and used the Hardin-Shumway test (Supplement Methods) (Hardin and Shumway, 1996) to examine whether there was a statistically significant difference between the two sex-specific BAcc scores. The second approach was a General Linear Model that regressed the prediction scores of the 106 USYA participants from sex, drinking group, and their interaction. A significant interaction would indicate that the gap in prediction scores between heavy and non-heavy drinkers was significantly different by sex. A significance threshold of p<0.05 was used in all statistical tests.

Next, we used the SHapley Additive exPlanations (SHAP) value (Sundararajan and Najmi, 2020) to identify a subset of factors that contributed the most to the classification (Supplement Methods). For each of those identified factors, a t-test examined its group difference between the heavy and non-heavy drinkers. For factors with significant group differences (p<0.05 after correcting for the number of identified factors with the Bonferroni procedure), the t-test was separately repeated within each sex. Lastly, the identified factors by SHAP were compared to the factors selected by a LASSO analysis.

We selected a subset of 106 4YCP participants by matching their race, recruitment site, and socioeconomic status with the 106 USYA participants (Supplement Methods), as compared with the 106 USYA participants, the 453 4YCP individuals were already matched with respect to sex (p>0.1, $\chi^{2}$ test) but had more African-American participants (p<0.001, $\chi^{2}$ test), were more often recruited at the University of California San Diego site (p=0.021, $\chi^{2}$ test), and had lower socioeconomic status (p<0.001, two-sample *t*-test) as measured by the highest years of education of either parent (Table 1). Then, procedures of cross-validating the classification model, analyzing sex-specific classification accuracy, and computing SHAP values for individual factors were repeated on this matched 4YCP subset.

## 3 Results

### 3.1 Trajectory of alcohol use in 4YCP and USYA individuals

**Fig. 2:**
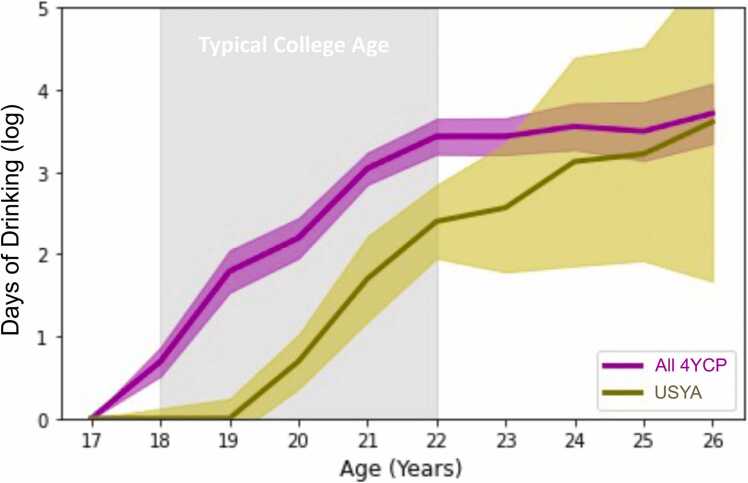
Log of annual days of drinking over age for all 453 4YCP participants and 106 USYA participants. The two center curves indicate the median at each age interval, and the two bands show the confidence interval of the median. While 4YCP participants drank heavier during the college years, the USYA cohort caught up later.

Confined to the 559 participants who were no-to-low drinkers before age 18 years, 28 % of the 106 USYA participants initiated heavy drinking during young adulthood. This ratio was significantly lower than that of the 4YCP participants (57 %, $\chi_{1,N=559}^{2}=28.2,\;$p<0.001). According to a mixed effect model (Supplement Table S1), USYA individuals also consumed significantly less alcohol (p<0.001 for college group difference) at or after age 18 years compared to the 4YCP cohort. Also significant was the age-by-college-group interaction (p<0.001), indicating that the difference in alcohol use between 4YCP and USYA was larger in the typical age range of 4-year residential college students (i.e., 18–22 years) than the difference after age 22 years (Fig. 2). Repeating the mixed effect model by replacing the days of drinking with days of tobacco use and cannabis use (Supplement Figure S1) revealed that there was no significant difference in use of those substances during the typical college age (18–22 years) between USYA and 4YCP participants, but the difference emerged thereafter (after age 22 years).

### 3.2 Prediction of initiating heavy drinking in young adulthood

**Fig. 3:**
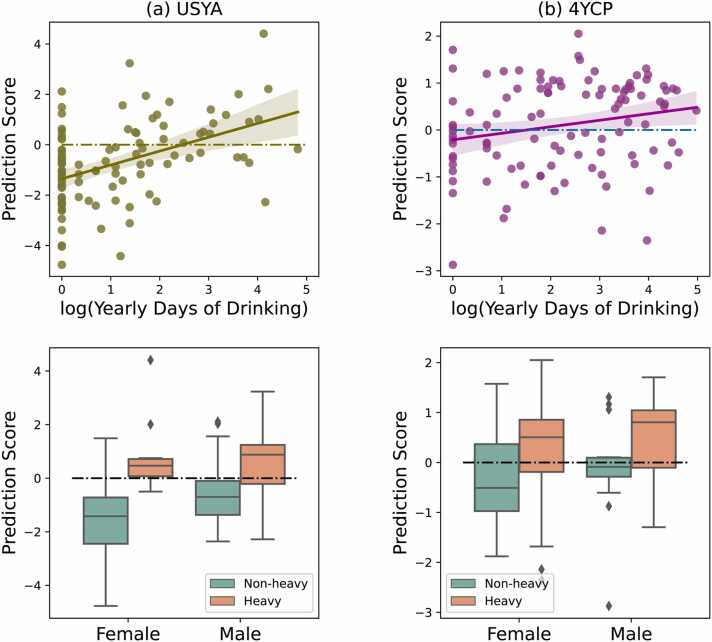
Top row: Correlation between SVM prediction scores and average yearly alcohol consumption (log-scaled) at or after 18 years for (a) 106 USYA and (b) 106 4YCP participants. Bottom row: Distribution of SVM prediction scores of males and females in the 106 USYA and 106 4YCP participants. Dashed lines indicate the threshold of the machine learning model for classifying heavy vs. non-heavy drinkers.

Cross-validating the classifier on the USYA cohort resulted in a 78.8 % accuracy (BAcc: 78.8 %, AUC: 80.3 %, and F1: 77.6 %, Table 2) in classifying those who initiated heavy drinking at or after age 18 years versus those who did not. The classification accuracy for the 106 demographic-matched 4YCP participants (BAcc: 66.8 %) was lower than the one for USYA (Hardin-Shumway test, p<0.001, Table 2) but significantly better than chance ($\chi_{1,N=106}^{2}=12.2$, p<0.001). For both 4YCP and USYA classification, the continuous prediction scores of the participants significantly correlated with their average yearly days of drinking at age 18 years or older (*p*<0.05, Pearson’s Correlation, Fig. 3 top row) and was uncorrelated with their baseline age.

**Table 2:** Accuracy of forecasting participants who initiated heavy drinking at or after age 18 year.

**Participants**	**N** **(non-Heavy/Heavy)**	**BAcc** **(%)**	**AUC** **(%)**	**F1** **(%)**
**USYA**	106 (76/30)	78.8	80.3	77.6
Male	49 (29/20)	72.9	73.3	72.1
Female	57 (47/10)	84.7	85.7	83.9
**4YCP**	106 (44/62)	66.8	67.7	67.1
Male	38 (14/24)	67.6	65.2	68.6
Female	68 (30/38)	66.2	67.8	66.1

### 3.3 Identifying factors contributing to the prediction

**Fig. 4:**
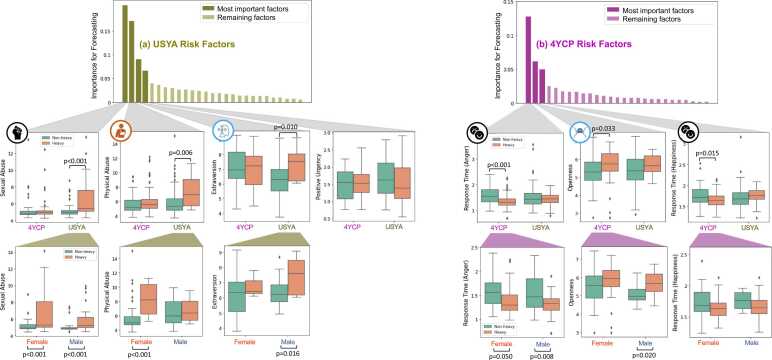
Top row: Factors ranked according to their importance (SHAP values) for the classification of non-heavy vs. heavy drinkers in the USYA and 4YCP cohort. Middle row: For the most important factors, the boxplots show the distribution of their values among the USYA and 4YCP participants. Factors with symbols correspond to the ones in Fig. 1, which indicate significant difference between the two drinking groups only within the USYA cohort or only within 4YCP. Bottom row: Factors significantly different between drinking groups are further plotted with respect to sex. The most important predictor for each cohort (i.e., sexual abuse for USYA and emotion identification time for 4YCP) is significantly different between drinking groups regardless of sex, while the other significant differences are sex-specific.

Specific to the USYA participants, the SHAP analysis identified 4 out of the 27 factors assessed before age 18 years that contributed the most to the classification of heavy vs. non-heavy drinking at or after 18 years (Fig. 4a). Three of these factors also differentiated the groups based on univariate *t*-tests (p<0.0125 after correcting for multiple comparisons) with USYA heavy drinkers having more childhood physical and sexual abuse and higher extraversion scores than the USYA non-drinkers. None of the 4 factors forecasting drinking identified in the USYA cohort showed significant group differences between drinkers and non-drinkers in the matched 4YCP cohort.

Lastly, repeating the SHAP analysis on the matched 4YCP cohort revealed that 3 out of the 27 factors contributed the most to the classification (Fig. 4b), where 4YCP heavy drinkers had faster response time (for the correct identifications) in identifying happiness and anger emotions (p<0.017 after correcting for multiple comparisons) and higher openness trait than 4YCP non-heavy drinkers (p=0.033, trend-level significance). For both USYA and 4YCP analysis, the identified factors agreed with those selected by the LASSO analysis (Supplement Results).

### 3.4 Risk factors for predicting drinking are sex-linked

The classification accuracy did not differ between male and female 4YCP participants (male 67.6 % vs. female 66.2 %), but differed in USYA participants, where the classification accuracy for female USYA (84.7 %) was significantly higher (*p*=0.041, Hardin-Shumway test) than for male USYA (72.9 %) (Table 2). This finding was further supported by a General Linear Model test identifying a drinking by sex interaction on the prediction scores of USYA participants, indicating that the gap between female heavy drinkers and female non-heavy drinkers was significantly larger than the gap for male participants (*p*=0.048, Fig. 3 bottom row). Other than this sex-linked difference, there was no significant difference in accuracy scores (BAcc p>0.05, Hardin-Shumway test) between other subcohorts, i.e., between participants who did and did not use tobacco or cannabis at age 18 years or older, or between the participants who enrolled in community colleges and those who did not go to college (Supplement Table S2).

For each of the risk factors identified by the SHAP analysis, sex-specific t-tests among the USYA participants revealed that history of physical abuse was greater in female drinkers (p<0.001) than in female non-drinkers but did not differ between the two male groups. Further, extraversion scores were higher in male drinkers than non-drinkers (p=0.016) but did not differ between the two female groups. History of sexual abuse was greater in heavy drinkers than non-heavy drinkers in both sexes (Fig. 4a, p<0.001). Among the 4YCP participants (Fig. 4b), openness was higher in male heavy drinkers than male non-drinkers (p=0.020), and the response time of emotion identification in heavy drinkers was faster than non-heavy drinkers regardless of sex (p<0.05).

## 4 Discussion

Our study is unique in using machine learning to identify risk factors in no-to-low drinking high school students that forecast drinking onset post high school in youth who made different post-high school career choices. Our analysis revealed that factors predicting heavy drinking onset for USYA individuals included childhood sexual and physical abuse (among female drinkers) and the trait of extraversion (among male drinkers) during high school. These risk factors were different from the ones for the commonly studied 4YCP participants, which included greater openness (specific to male youth) and ability to recognize facial emotions measured in high school. Collectively, these results indicate a divergence of predictors of heavy alcohol use in youth depending on post-high school career choice.

Whereas some research on alcohol use during college grouped young adults attending any type of college (Lee et al., 2020, McCabe et al., 2021), our cohort design was motivated by findings suggesting that alcohol-related behavior in young adults attending community colleges is similar to those not attending college (Timberlake et al., 2007) and significantly different from 4YCP individuals (Duckworth et al., 2022, Velazquez et al., 2011). This distinction between types of college may be driven by contextual influences associated with college life (Cleveland et al., 2018). For example, living without parental supervision and within proximity to a large cohort of peers, factors likely to be specific to 4YCP attending residential colleges, may encourage social processes and peer pressures that lead to excessive alcohol consumption (Carter et al., 2010).

Our analysis also revealed that the predisposing effects during high school were more informative for forecasting drinking onset in USYA than 4YCP individuals. This difference in prediction accuracy may also depend on the onset time of heavy drinking. 4YCP individuals were more likely to initiate heavy drinking during ages typical for college, which was averted by their USYA peers (Fig. 2) living in different social environments, parental supervision, and work responsibilities and demands (Butler et al., 2010, White and Hingson, 2013). The riskier consumption patterns among 4YCP individuals exposed them to an increased risk of experiencing negative physical, academic, and interpersonal consequences during the typical college age (Ham and Hope, 2003, SAMHSA, 2020). These results point to the need to enhance prevention and intervention in residential colleges to educate students about the adverse effects of illegal underage drinking behavior.

Past studies identified numerous factors that influence the risk of alcohol use among adolescents and young adults, giving rise to highly heterogeneous developmental pathways to heavy drinking onset (Kenney et al., 2018). To this end, our analysis indicates that USYA individuals were associated with unique risk factors recorded during high school that forecast later heavy drinking onset. Specific to the USYA prediction was childhood trauma (sexual and physical abuse), which has been frequently linked to early onset of substance use (Carliner et al., 2016, Clark et al., 2010) and other psychiatric problems related to anxiety and depression during adolescence (Li et al., 2022, Tong et al., 2022). In addition, exposure to traumatic events often exerts a negative influence on academic performance during high school, lead to more school absences, and increase the possibility of dropping out of school (APA, 2021). As a result, childhood trauma experiences may become a more salient risk factor for later onset heavy drinking in young adulthood among those who do not attend 4-year colleges.

On the other hand, the compromised ability of emotion identification during adolescence was a risk factor for future heavy drinking onset in the 4YCP cohort (Davis et al., 2022). Although the mechanism underpinning alcohol-related deficits in emotion differentiation was unclear, poorer emotion recognition ability had been identified as a risk factor predicting drinking relapse in AUD treatment (Emery et al., 2022, Rupp et al., 2017). In our study, the altered emotion recognition was characterized as a faster (rather than slower) emotion identification of anger and happiness among heavy drinking 4YCP participants than non-heavy drinkers. A post-hoc analysis indicates that the speeded responses were also accompanied by significantly fewer correct identifications and a higher false positive rate among 4YCP drinkers (Supplement Figure S2). This shift in “speed-accuracy tradeoff” with low accuracy and high speed of performance has traditionally been understood as an effect of alcohol use disorder in adults (Glenn and Parsons, 1991). Critically, our analysis suggests it should also be recognized as a phenomenon pre-existing drinking and highlights the translational value of emotion recognition capability that is often overlooked by current preventive programs.

In addition to post-high school career choices, male and female youth showed unique neurobiological and behavioral sex risk factors during high school for future drinking onset. The higher forecasting accuracy in girls than boys (Table 2) further substantiates the result that female youth in the USYA cohort were associated with stronger predisposing effects of alcohol use than their male counterparts. In this cohort, that observed difference appeared to be fostered by childhood physical abuse among the female youth (Fig. 4). Although boys are more likely to have experienced physical abuse, girls tend to have more negative consequences from physical abuse, including having a greater risk of alcohol abuse or dependence later in life (Thompson et al., 2004). Common among male youth, personality traits were identified as male-specific risk factors in both 4YCP and USYA analyses, with 4YCP male drinkers having greater openness and USYA male drinkers having greater extraversion than the non-drinkers. Given the well-established sex differences in these personality characteristics in the general population (Weisberg et al., 2011), our results further suggest the potential role of sex in modulating the personality profile and forming sex-specific pathways to heavy drinking in USYA.

There were a few notable limitations in our study. Frist, the sample size was bounded by the number of USYA participants in the NCANDA cohort, which was under-powered to further subtype risk factors specific to detailed career choices (e.g. college dropout, military, workforce). The limited number of female heavy drinkers in USYA precluded investigating sex differences in a wider range of risk factors (e.g., neuroimaging measurements) collected by NCANDA. Thus, replication analysis on larger datasets is needed to assess the current findings. Lastly, our analysis focused only on participants who were no-to-low drinkers before age 18 years. The drinking trajectories and factors for forecasting drinking patterns for those who already initiated drinking during adolescence might be different and require further investigation.

## 5 Conclusion

We present the first study to forecast heavy drinking among young adults based on mental-health risk factors recorded from them in high school before initiating appreciable drinking. Notably, risk factors for drinking onset differed by sex and post-high school career choices. These results highlight the need to develop prevention programs that are unique to understudied young adults, i.e., those joining the workforce, military, or community colleges, who represent the majority in this age cohort.

## Funding

This work was supported by the U.S. National Institute on Alcohol Abuse and Alcoholism [AA010723 (EVS), AA028840 (QZ), AA021697 (AP+KMP), AA021695 (SFT+SAB), AA021692 (SFT), AA021696 (MDZ+FCB), AA021681 (MDDB), AA021690 (DBC), AA021691 (BJN)] with co-funding from the 10.13039/100000026National Institute on Drug Abuse [DA057567 (KMP+ST)], the 10.13039/100000025National Institute of Mental Health, and the 10.13039/100000071National Institute of Child Health and Human Development. The work was also supported by BBRF NARSAD Young Investigator Grant (QZ), the Daegu Gyeongbuk Institute of Science & Technology (DGIST) Joint Research Project (KMP), and HAI-Google Cloud Credits Award (KMP). Funders had no role in design and conduct of the study; collection, management, analysis, and interpretation of the data; preparation, review, or approval of the manuscript; and decision to submit the manuscript for publication.

## CRediT authorship contribution statement

**Adolf Pfefferbaum:** Writing – review & editing, Methodology, Funding acquisition. **Bonnie Nagel:** Writing – review & editing, Funding acquisition. **Beatriz Luna:** Writing – review & editing, Funding acquisition. **Susan Tapert:** Writing – review & editing, Funding acquisition. **Magdalini Paschali:** Methodology. **Sandra Brown:** Writing – review & editing, Funding acquisition. **Qingyu Zhao:** Writing – original draft, Validation, Methodology, Formal analysis. **Kilian Pohl:** Writing – review & editing, Validation, Methodology, Funding acquisition, Data curation, Conceptualization. **David Goldston:** Writing – review & editing, Funding acquisition. **Edith Sullivan:** Writing – review & editing, Supervision, Funding acquisition, Conceptualization. **Michael De Bellis:** Writing – review & editing, Funding acquisition. **Duncan Clark:** Writing – review & editing, Funding acquisition. **Kate Nooner:** Writing – review & editing, Funding acquisition. **Wesley Thompson:** Writing – review & editing, Funding acquisition. **Fiona Baker:** Writing – review & editing, Funding acquisition. **Sonja Eberson:** Project administration, Data curation. **Joseph Dehoney:** Data curation. **Massimiliano de Zambottib:** Writing – review & editing, Funding acquisition.

## Declaration of Competing Interest

None of the authors have biomedical financial interests or conflicts of interest with the reported data or their interpretation.

## Data Availability

The data were based on a formal, locked data release NCANDA_NIAAADA_BASE_V01, NCANDA_NIAAADA_01Y_V01 to NCANDA_NIAAADA_08Y_V01 available via http://nda.nih.gov/edit_collection.html?id=4513.
