# Supplementary material for: Identifying high school risk factors that forecast heavy drinking onset in understudied young adults
Source: Dev Cogn Neurosci. 2024 Jun 26;68:101413. doi: 10.1016/j.dcn.2024.101413 (PMC11261404; doi:10.1016/j.dcn.2024.101413)
Supplement: Supplementary file 1 — Supplementary material [file mmc1.docx]

**Supplemental Materials for**

**Identifying High School Risk Factors that Forecast**
**Heavy Drinking Onset in Understudied Young Adults**

Qingyu Zhao^a^, PhD, Magdalini Paschali^b^, PhD, Joseph Dehoney^c^, BS,

Fiona C. Baker^c^, PhD, Massimiliano de Zambotti^c^, PhD, Michael D. De Bellis, MD, MPH^d^, David B. Goldston^d^, PhD, Kate B. Nooner^e^, PhD, Duncan B. Clark^f^, MD, PhD, Beatriz Luna^f^, PhD, Bonnie J. Nagel^g^, PhD, Sandra A. Brown^h,i^, PhD,

Susan F. Tapert^i^, PhD, Sonja Eberson^i^, MA, Wesley K. Thompson^j^, PhD, Adolf Pfefferbaum^c,k^, MD,

Edith V. Sullivan^k^, PhD, Kilian M. Pohl^c,k^*, PhD

**Affiliations:**

^a^Department of Radiology, Weill Cornell Medicine, New York, NY,

^b^Department of Radiology, Stanford University, Stanford, CA,

^c^Center for Health Sciences, SRI International, Menlo Park, CA, USA.

^d^Department of Psychiatry and Behavioral Sciences, Duke University School of Medicine, Durham, NC, USA

^e^Department of Psychology, University of North Carolina Wilmington, Wilmington, NC, USA.

^f^Department of Psychiatry, University of Pittsburgh, Pittsburgh, PA, USA

^g^Departments of Psychiatry and Behavioral Neuroscience, Oregon Health & Science University, Portland, OR, USA

^h^Department of Psychology, University of California San Diego, La Jolla, CA, USA

^i^Department of Psychiatry, University of California San Diego, La Jolla, CA, USA

^j^Laureate Institute for Brain Research, Tulsa, OK, USA

^k^Department of Psychiatry and Behavioral Sciences, Stanford University, Stanford, CA

*Address correspondence to Kilian M. Pohl, Department of Psychiatry and Behavioral Sciences, Stanford University School of Medicine, 401 Quarry Rd., Stanford, CA, 94305, USA, United States. Email: [kilian.pohl@stanford.edu](mailto:kilian.pohl@stanford.edu)

# Supplement Methods

**Participants.** The NCANDA cohort [1] comprises 831 participants, ages 12 to 21 years at baseline who were recruited across five collection sites: University of California San Diego, SRI International, Duke University Medical Center, University of Pittsburgh, and Oregon Health & Science University and assessed yearly on psychobiological measures. The Institutional Review Boards (IRB) of each site approved the standardized data collection and use [1]. Of the 831 participants, 23 were excluded from current analyses due to aberrancies noted in neuroradiology reports at their baseline visits [2]. The cohort definition of USYA aligns with the NIAAA Notice of Special Interest (NOSI): Epidemiology and Prevention of Alcohol Misuse in Understudied Young Adult Populations; Military, Workforce, and Community College, which defines USYA as persons aged 18 to 29 who are not enrolled in four-year colleges or universities. Based on the above criteria, 752 participants had baseline visits before age 18 years and known college status. Further excluded in our analysis were 193 individuals who started drinking before age 18 years, of whom 159 went to 4-year colleges and 34 did not.

**Drinking Criteria.** Based on self-reported alcohol use history (Customary Drinking and Drug Use Record and Time Line Follow Back [1]) collected throughout the first 8 years of the study, drinking levels of participants were defined based on the youth-adjusted Cahalan score on a scale of 0 to 3, which considered quantity and frequency of past-year drinking patterns [3, 4]. Heavy drinkers ranged from moderate frequency (e.g., 2x/month) with high quantity consumption (e.g., with 3-4 drinks on average and > 4 drinks maximum) to higher frequency (e.g., 1x/week or more) with moderate quantity consumption (e.g., with 2-3 drinks on average and >4 drinks maximum).

**Mental Health Measurements.** History factors of a participant included family drinking history and self-reported traumatic experiences captured by the Childhood Trauma Questionnaire [5], which consists of five subscales: physical, emotional, and sexual abuse, and emotional and physical neglect, with higher scores corresponding to greater traumatic experience. Depressive symptoms were measured by the Center for Epidemiologic Studies Depression Scale (CES-D-10 [6]) score, which ranges from 0 to 30, with higher scores indicating the presence of more depressive symptoms. Personal traits were captured by the UPPS-P Impulsive Behavior Scale [7] and the Ten-Item Personality Inventory [8]. UPPS-P consists of 20 statements (scale of 1-4) examining 5 impulsivity sub-scales measuring urgency, lack of premeditation, perseverance, sensation seeking, and positive urgency. The TIPI assesses broad personality domains of conscientiousness, agreeableness, extraversion, emotional stability, and openness to experiences. Neuropsychological scores were assessed by the Stroop task, the Penn Emotion Recognition Test, and the Short Fractal N-back Test. The Stroop Match-to-Sample task [9] was a computerized task of executive control assessing component processes of cognitive control (Stroop color-word interference) and motor control (response selection). The task required adolescents to match the color of a sample stimulus to the color of a Stroop word and press a “Yes” key, if the word’s font color matches with the initially presented sample’s color and press a “No” key when the sample and the word’s font color do not match. In the current analysis we included the overall mean and standard deviation of the response time. The Penn Emotion Recognition Test [10] measures the ability to identify six basic emotions—angry, scared, happy, sad, and neutral—in facial expressions displayed on a screen. The task required participants to indicate the expressed emotion from a list of 5 choices by clicking the word describing the emotion, one at a time. In our analysis, we used the median response time for the correct identifications of the 5 emotions. Lastly, attention and working memory were measured by the Short fractal N-back test [11]. In the 0-back condition, the target was a fractal design displayed on the computer screen that matched a prespecified fractal image. In the 1-back condition, the participants had to identify temporally adjacent repetitions. The target was any pattern identical to the design presented one trial back. In the 2-back condition, the target was any design that was identical to the one presented two trials back. We used the true positive rate and median response time for all correct responses in the test.

**Removing Confounding Effects from Factors.** For each of the 27 mental health factors, a linear mixed effects model was fitted on all 559 participants who were no-to-low drinkers before age 18 years by regressing the factor value of each visit from age of each visit, sex, ethnicity, site, and socioeconomic status. Then for each visit of a participant, the influence from those covariates was residualized from that factor. The residualized values over all visits of a participant before age 18 years were averaged. In the end, each participant was described by a 27-dimensional feature vector.

**Data Matching.** A demographically matched data set of 4YCP and USYA individuals was created using the maximum bipartite matching algorithm [12, 13]. The method first constructed a bipartite graph such that the first set of nodes represented 453 4YCP participants and the second set of nodes represented 106 USYA participants. An edge was connected between a 4YCP and a USYA participant if they were of the same site, race, and had a gap of socioeconomic status less than or equal to 1. A Ford-Fulkerson algorithm [14] was then applied to select a maximum number of matching pairs. This process resulted in a matched dataset of 106 USYA and 106 4YCP individuals.

**Evaluation Metrics for Classification.** Balanced accuracy (BAcc) [15] is a commonly used metric for evaluating how good a binary classifier is. It is the arithmetic mean of sensitivity (true positive rate) and specificity (true negative rate) of the classification outcome. It is especially useful when the classes are imbalanced, i.e. one class having more samples than the other. Also robust to class imbalance is the area under a receiver operating characteristic (ROC) curve, known as AUC [16]. It is a scalar metric that measures the overall performance of a binary classifier involving all possible classification thresholds. The value of AUC is between 0.5 and 1.0, where 0.5 represents the performance of a random classifier and 1.0 corresponds to a perfect classifier. A third metric for measuring a classifier’s accuracy while being impartial to class imbalance is F1-score [17], computed as the harmonic mean of precision and recall. A model will obtain a high F1 score only if both precision and recall are high.

**Confidence Scores of the SVM Classifier.** Support Vector Machines (SVMs) are a non-probabilistic binary linear classifier. It seeks the best hyperplane in the high-dimensional feature space that represents the largest separation, or margin, between the data points of the two classes (i.e., the distance from the hyperplane to the nearest data point on each side is maximized). In our study, we followed the explanation provided by the *sklearn* package [18] to define the “confidence” score for the classification of a testing data point as the signed distance of that sample to the hyperplane (the sign denotes which class it is assigned to). Note, as the SVM is not a probabilistic model, these confidence scores do not directly translate to real probability measures.

**Filling in Missing Feature Values.** The average missing rate across the 27 features was 27.6%, with all participants having family history information and 33% of the participants missing the Stroop test. Missing values were imputed by the k-nearest-neighbor algorithm implemented in Matlab R2021b (i.e., *knnimpute*) [19]. As this imputation step did not use the label of drinking groups (unsupervised learning), it would not bias the results of the following supervised machine learning by SVM.

**Hardin-Shumway Test for Comparing Balanced Accuracy Scores.** In [20], Hardin and Shumway proposed to a non-parametric way to test the statistical significance of the difference between two classification accuracy scores A and B. Assume the accuracy score A can be computed from D $=\left\{ <g_{i},p_{i}> \right|i=1,\ldots,m\}$ produced by a machine learning model, where $g_{i}$ is the ground-truth label of the $i^{th}$ sample and $p_{i}$ is the predicted label by the model. We can derive a new score A’ from a bootstrapped D’, i.e., by sampling from D with replacement for $m$ times. Repeating the bootstrapping procedure for a large number of times (K=5000) gives rise to the empirical null distribution of A, i.e., {A’_1, …,_ A’_5000_}. The p-value of B being significantly larger than A is the proportion of the sampled A’_j_ that is larger the B.

**SHAP Analysis.** In each training run of the Leave-One-Out cross-validation, the *shapley* function implemented in Matlab R2021b was applied to the trained model and the one test sample, which produced 27 SHAP values quantifying the contribution of each factor for classifying that test case. The final SHAP value associated with a factor is the absolute value of the average SHAP values over the 106 training runs. To identify a subset of factors of high contribution, a piecewise linear model was fitted to the sorted SHAP values. The model considered two linear segments and used the `residual sum of squares’ to automatically determine the breakpoint [21]. Finally, the first segment before the break point contained factors with greatest contribution to the model.

**LASSO Classification**: To examine whether the identified factors were dependent on the choice of classifiers, we repeated the Leave-One-Out cross-validation by replacing the SVM classifier with the LASSO classifier. The BAcc was evaluated with different choices of the regularization weight $\lambda$ = {0.0001, 0.001, 0.01, 0.1, 1}. To quantify the importance of each factor, the linear coefficients of the trained LASSO model [22] were averaged across cross-validation runs and ranked according to their magnitude.

# **Supplement Results**

**LASSO Classification:** The highest BAcc for USYA was 70.2% with $\lambda=0.01$ and 64.9% for 4CYP with the same $\lambda=0.01$. After ranking the contribution of the factors, the top 3 factors for 4YCP were response time to anger, response time to happiness, and openness. The top 4 factors for USYA were extraversion, sexual abuse, physical abuse, and positive urgency. Despite the different rank among the factors, the top factors identified by LASSO were the same as revealed by SVM and SHAP analysis.

**Table S1**. Results of the mixed effects model applied to the annual log of days of drinking of 453 4YCP participants and 106 USYA participants. Significant fixed effects (p<0.05) are typeset in bold.

|  | Estimate | SE | tStat | p-value |
| --- | --- | --- | --- | --- |
| Intercept | **-31.558** | **1.223** | **-25.793** | **<0.001** |
| age | **2.665** | **0.105** | **25.323** | **<0.001** |
| age^2^ | **-0.053** | **0.002** | **-21.844** | **<0.001** |
| age:college | **-0.115** | **0.024** | **-4.816** | **<0.001** |
| college | **1.849** | **0.502** | **3.683** | **<0.001** |
| sex | 0.046 | 0.098 | 0.475 | 0.635 |
| Site-UPMC | 0.201 | 0.155 | 1.298 | 0.194 |
| Site-SRI | **0.565** | **0.163** | **3.463** | **<0.001** |
| Site-Duke | **0.451** | **0.147** | **3.064** | **<0.001** |
| Site-OHSU | 0.091 | 0.143 | 0.635 | 0.525 |
| Race-Caucasian/white | 0.210 | 0.248 | 0.847 | 0.397 |
| Race-African-American | -0.086 | 0.286 | -0.300 | 0.765 |
| Race-Asian | -0.279 | 0.291 | -0.958 | 0.338 |
| Socioeconomic status | **0.051** | **0.022** | **2.304** | **0.021** |

**Table S2**. Classification accuracy within subgroups of USYA and 4YCP participants. Subgroups of substance use were determined by the median of annual days of tobacco or cannabis use after age 18 years.

|  | **Subgroups** | **N**  **(Non-Heavy / Heavy)** | **BAcc %** | **AUC %** | **F1 %** |
| --- | --- | --- | --- | --- | --- |
| **USYA** | **College Subgroups** |  |  |  |  |
|  | Community College | 67 (47/20) | 78.1 | 78.4 | 76.5 |
|  | Non-College | 39 (29/10) | 80.6 | 86.3 | 80.0 |
|  | **Cannabis-use Subgroups** |  |  |  |  |
|  | yearly use = 0 | 53 (43/7) | 78.1 | 72.7 | 76.5 |
|  | yearly use > 0 | 53 (30/23) | 78.6 | 82.6 | 77.6 |
|  | **Tobacco-use Subgroups** |  |  |  |  |
|  | yearly use = 0 | 73 (62/11) | 82.0 | 80.9 | 82.0 |
|  | yearly use > 0 | 33 (14/19) | 80.6 | 84.6 | 77.9 |
| **4YCP** | **Cannabis-use Subgroups** |  |  |  |  |
|  | yearly use = 0 | 55 (33/22) | 69.7 | 66.1 | 70.6 |
|  | yearly use > 0 | 51 (14/37) | 67.7 | 65.6 | 63.7 |
|  | **Tobacco-use Subgroups** |  |  |  |  |
|  | yearly use = 0 | 57 (33/24) | 66.7 | 66.9 | 66.7 |
|  | yearly use > 0 | 49 (11/38) | 66.0 | 71.8 | 66.8 |

**Table S3**. Demographic information of the USYA cohort, i.e., NCANDA participants who enrolled in community colleges or did not go to any type of college (non-college).

|  | **Community College** | **Non-College** |
| --- | --- | --- |
| **N (M/F)** | 67 (31/36) | 39 (18/21) |
| **Age at baseline** | 16.1$\pm$2.5 | 15.0$\pm$2.4 |
| **Number of visits** | 7.2$\pm$1.8 | 7.0$\pm$1.7 |
| $\boldsymbol{\dagger}$**Race (%)** |  |  |
| Caucasian | 44 (66%) | 17 (43%) |
| African-American | 18 (27%) | 16 (41%) |
| Asian | 2 (3%) | 3 (8%) |
| Others | 3 (4%) | 3 (8%) |
| $\boldsymbol{\dagger}$**Site (%)** |  |  |
| UPMC | 11 (16%) | 7 (18%) |
| SRI | 7 (10%) | 1 (3%) |
| Duke | 9 (13%) | 12 (31%) |
| OHSU | 12 (18%) | 10 (26%) |
| UCSD | 28 (42%) | 9 (23%) |
| **SES** | 14.9$\pm$2.7 | 14.4$\pm$2.3 |

**
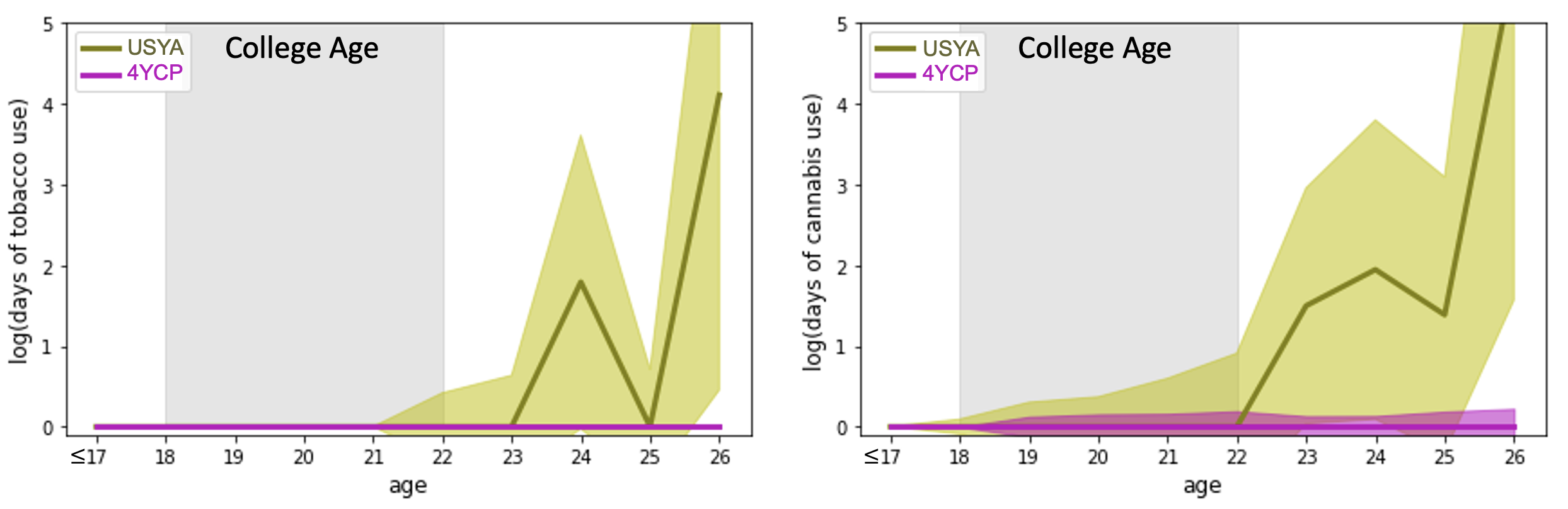
Figure S1**. Log of annual days of tobacco use (left) and cannabis use (right) over age for the 453 4YCP participants and 106 USYA participants. Two center curves indicate the median at each age interval, and the two bands show the confidence interval of the median.


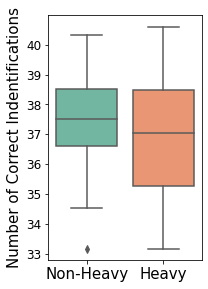


**Figure S2**. Number of correction identifications in the Penn Emotion Recognition Test for the 4YCP cohort.

**References**

p=0.007

p=0.001

p=0.479

p=0.009

1. Brown, S.A., et al., *The National Consortium on Alcohol and NeuroDevelopment in Adolescence (NCANDA): A Multisite Study of Adolescent Development and Substance Use.* J Stud Alcohol Drugs, 2015. **76**(6): p. 895-908.

2. Pfefferbaum, A., et al., *Adolescent Development of Cortical and White Matter Structure in the NCANDA Sample: Role of Sex, Ethnicity, Puberty, and Alcohol Drinking.* Cereb Cortex, 2016. **26**(10): p. 4101-21.

3. Pfefferbaum, A., et al., *Altered Brain Developmental Trajectories in Adolescents After Initiating Drinking.* Am J Psychiatry, 2018. **175**(4): p. 370-380.

4. Cahalan, D., I.H. Cisin, and H.M. Crossley, *American drinking practices: A national study of drinking behavior and attitudes.* *Monographs of the Rutgers Center of Alcohol Studies*, 1969. **6**(260).

5. Bernstein, D.P., et al., *Initial reliability and validity of a new retrospective measure of child abuse and neglect.* Am J Psychiatry, 1994. **151**(8): p. 1132-6.

6. Radloff, L.S., *CES-D scale: A self report depression scale for research in the general populations.* Applied Psychological Measurement, 1977. **1**: p. 385-401

7. Cyders, M.A., et al., *Examination of a short English version of the UPPS-P Impulsive Behavior Scale.* Addict Behav, 2014. **39**(9): p. 1372-6.

8. Nunes, A., et al., *Short Scales for the Assessment of Personality Traits: Development and Validation of the Portuguese Ten-Item Personality Inventory (TIPI).* Front Psychol, 2018. **9**: p. 461.

9. Schulte, T., et al., *Effects of age, sex, and puberty on neural efficiency of cognitive and motor control in adolescents.* Brain Imaging Behav, 2020. **14**(4): p. 1089-1107.

10. Gur, R.C., et al., *A method for obtaining 3-dimensional facial expressions and its standardization for use in neurocognitive studies.* J Neurosci Methods, 2002. **115**(2): p. 137-43.

11. Ragland, J.D., et al., *Working memory for complex figures: an fMRI comparison of letter and fractal n-back tasks.* Neuropsychology, 2002. **16**(3): p. 370-9.

12. Gibbons, A., *Algorithmic Graph Theory*. 1985: Cambridge University Press.

13. Rosenbaum, P.R., *Optimal Matching for Observational Studies.* Journal of the American Statistical Association, 1989. **84**(408): p. 1024–1032.

14. Ford, L.R. and D.R. Fulkerson, *Maximal flow through a network.* *Canadian Journal of Mathematics*, 1956. **8**: p. 399-404.

15. Park, S.H., et al., *Alcohol use effects on adolescent brain development revealed by simultaneously removing confounding factors, identifying morphometric patterns, and classifying individuals.* Sci Rep, 2018. **8**(1): p. 8297.

16. Hanley, J.A. and B.J. McNeil, *The meaning and use of the area under a receiver operating characteristic (ROC) curve.* Radiology, 1982. **143**(1): p. 29-36.

17. Goutte, C. and E. Gaussier, *A Probabilistic Interpretation of Precision, Recall and F-Score, with Implication for Evaluation*, in *European Conference on Information Retrieval*. 2005. p. 345–359.

18. *Linear Support Vector Classification*. Available from: <https://scikit-learn.org/stable/modules/generated/sklearn.svm.LinearSVC.html>.

19. *knnimpute*. Available from: <https://www.mathworks.com/help/bioinfo/ref/knnimpute.html>.

20. Hardin, P. and J.M. Shumway, *Statistical significance and normalized confusion matrices.* Photogramm. Eng. Remote Sens., 1996. **63**: p. 735 - 740.

21. McGee, V.E. and W.T. Carleton, *Piecewise Regression.* Journal of the American Statistical Association, 1970. **65**(331).

22. Friedman, J., T. Hastie, and R. Tibshirani, *Regularization Paths for Generalized Linear Models via Coordinate Descent.* J Stat Softw, 2010. **33**(1): p. 1-22.
